# Detecting Movement Changes in Children with Hemiparesis after Upper Limb Therapies: A Responsiveness Analysis of a 3D Bimanual Protocol

**DOI:** 10.3390/s23094235

**Published:** 2023-04-24

**Authors:** Marine Cacioppo, Mathieu Lempereur, Laetitia Houx, Sandra Bouvier, Rodolphe Bailly, Sylvain Brochard

**Affiliations:** 1 Department of Physical Medicine and Rehabilitation, Brest University Hospital, 29200 Brest, France; 2 Pediatric Rehabilitation Department, Fondation ILDYS, 29200 Brest, France; 3 Laboratoire de Traitement de L’Information Médicale (LaTIM), Inserm U1101, Université de Bretagne-Occidentale, 29200 Brest, France; 4 Pediatric Neurology Unit, Children’s Hospital, Geneva University Hospitals, 1205 Geneva, Switzerland

**Keywords:** children, cerebral palsy, motion analysis, upper limb, bimanual, kinematics, quality of movement, responsiveness, intensive motor therapy

## Abstract

The “Be an Airplane Pilot” (BE API) protocol was developed to evaluate upper limb (UL) kinematics in children with unilateral cerebral palsy (uCP) during bimanual tasks. The aim of this study was to investigate the responsiveness of this protocol to changes in kinematics and movement quality after UL therapies, using individual and group analyses, and to analyse the relationships between kinematic and functional changes in these children. Twenty children with uCP (5–15 years old) either participated in bimanual intensive therapy or received UL botulinum toxin injections. All the children performed the BE API protocol and functional assessments (Assisting Hand Assessment [AHA]) before and after the interventions. The individual analyses found kinematic changes in 100% of the children after therapy. The group analysis found significantly higher trunk and shoulder deviations after the intensive therapy. No significant changes were found for smoothness or trajectory straightness. The changes in the kinematic deviations were moderately correlated with the changes in the AHA scores. This study confirmed the responsiveness of the BE API protocol to change after therapy; therefore, the protocol is now fully validated and can be implemented in clinical practice. Its use should help in the accurate identification of impairments so that individualized treatments can be proposed.

## 1. Introduction

Difficulty performing bimanual tasks limits activities and participation in children with unilateral cerebral palsy (uCP) [1]. Interventions to prevent and manage upper limb (UL) impairments mainly include botulinum toxin injections (BoNT), surgery, casting, and an increasing number of training-based interventions, including bimanual training and constraint-induced movement therapy (CIMT). [2]. Studies have shown which treatments improve impaired UL use and which do not; however, the evidence is based on clinical measures.

The clinical measures of bimanual function involve video-based assessments or questionnaires (e.g., the Assisting Hand Assessment (AHA) [3], the ABILHAND-Kids [4]). These bimanual assessments are subjective or semi-quantitative, and the results are difficult to interpret, therefore, they do not provide enough help in terms of understanding the effect of therapies or determining individualized treatment programmes. Thus, an accurate and validated assessment of bimanual function that provides insights into abnormal movements and can objectively guide clinicians in the planning of impairment-based UL interventions is needed.

3D motion analysis is an objective method of movement assessment that is used to evaluate and monitor the effect of therapies that aim to improve lower limb function, such as BoNT or orthopaedic surgery. It has been demonstrated to influence decision-making [5]. Few intervention studies evaluating the effectiveness of therapy on UL movements using 3D motion analysis have been performed. Most studies used standardized unimanual tasks [6,7,8], and only a few have used bimanual assessments [9,10,11,12] that better reflect UL use in daily life.

In a recent systematic review of quantitative assessments of bimanual movements in children with CP, the authors reported that 3D protocols were heterogenous (1–5 tasks), and the drawer-opening task was the most studied but was not sufficiently representative of bimanual function [13]. The analyses have focused mostly on spatiotemporal variables, some of which were specifically developed to measure bimanual function (e.g., task completion time, goal synchronization). These instrumented measures have moderate to good validity, but their reliability and responsiveness have not been evaluated, limiting their use as references in clinical practice. Pre–post-intervention studies using the drawer-opening task demonstrated significant changes in bilateral upper limb function (spatiotemporal variables) and some kinematic changes after bimanual training; no changes were found after CIMT [9,11,14]. No other types of intensive motor therapy have been evaluated using instrumented measures, and no other variables have been used to evaluate change.

We developed a 3D bimanual protocol, “Be An Airplane Pilot” (BE API), to assess impaired UL movement in children with uCP using a set of five bimanual tasks performed within a game scenario [15,16]. The second version (BE API 2.0) of this protocol was the first to propose a comprehensive assessment of all the degrees of freedom (DoF) of the upper limb in bimanual conditions. This protocol was found to have good to excellent validity and reliability for kinematic and quality of movement variables in children with uCP. However, the responsiveness of the protocol has not yet been verified, i.e., its ability to detect a change in outcomes after a therapeutic intervention. This is necessary to ensure its utility for clinical practice. Furthermore, the relationship between changes in bimanual kinematics and changes in clinical scores after a therapeutic intervention has not yet been explored. This would increase understanding of the improvements in bimanual function that can be expected with treatment.

The first aim of this study was to compare the kinematic movement patterns (deviations and waveform analysis) of the impaired UL of children with uCP during bimanual tasks (BE API 2.0 protocol) before and after a therapeutic UL intervention (Hand and Arm Bimanual Intensive Therapy Including Lower Extremities [HABIT ILE] or botulinum toxin injections [BoNT]), using an individual analysis approach. The second aim was to compare the kinematic movement patterns of the impaired UL during bimanual tasks before and after HABIT ILE using a group analysis approach. The third aim was to compare the quality of movement variables (smoothness and trajectory straightness) of the impaired UL during bimanual tasks before and after the UL interventions using individual and group analyses. The fourth aim was to evaluate the relationship between changes in kinematic and quality of movement variable values for the trunk and the impaired UL and changes in functional assessment scores (bimanual performance: Assisting Hand Assessment; and unimanual capacity: Melbourne Assessment 2) after the UL interventions.

## 2. Materials and Methods

### 2.1. Ethical and Regulatory Considerations

This prospective study was approved by the Brest ethical committee (B2021CE.48). The assessments performed in this study were part of routine clinical practice. All the parents and children received verbal and written information about the study before participating and completed a non-opposition form.

### 2.2. Participants

Children with uCP/hemiparesis were recruited between July 2020 and July 2022 at Brest University Hospital. The inclusion criteria were children aged from 5 to 17 years with a sufficient grasp ability to perform the protocol tasks (Manual Ability Classification System [MACS] level I to III) and either participating in an intensive UL motor therapy programme (HABIT ILE) or with a planned BoNT injection. The exclusion criteria were severe cognitive or visual disturbances, UL pain, previous UL surgery, and botulinum toxin injections less than 3 months prior to participation.

### 2.3. Interventions

The children underwent one of two interventions: HABIT ILE or BoNT injections.

HABIT-ILE is based on the concepts of structured motor learning and intensive therapy [17]. This therapy improves UL motor performance in children with uCP [18] in the three domains of the International Classification of Functioning and Disability: anatomical structure and function (strength and dexterity); activity (activities of daily living); and participation (achievement of personal goals). The HABIT ILE camps included 65 h of bimanual therapy over 2 weeks and were performed as part of usual clinical practice.

Intramuscular injections of BoNT are used to reduce local spasticity. The injections were prescribed by the child’s referring pediatric physical and rehabilitation medicine physician, who defined the muscle sites to be injected and the doses according to current recommendations [19]. The injections were performed by two experienced physicians using ultrasound and/or electrostimulation guidance. After the BoNT injections, the child underwent physical therapy (two sessions per week for 4–6 weeks with home exercises) [2,20].

### 2.4. Study Schedule

**Figure 1:**
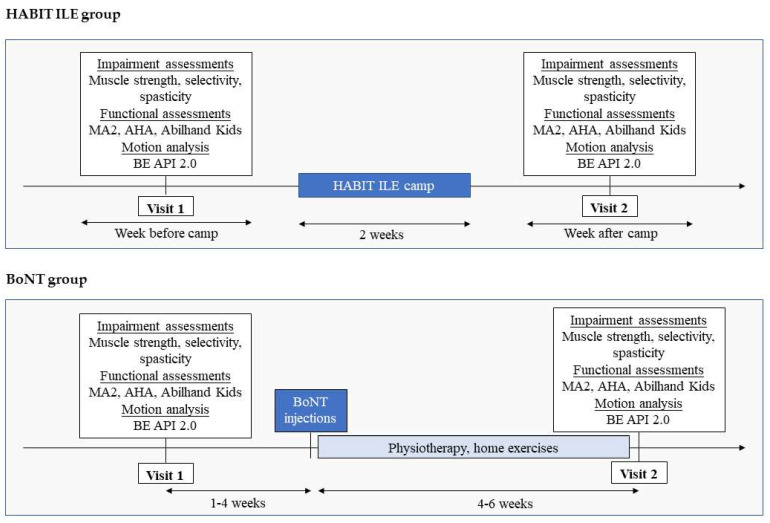
Study schedule. AHA: Assisting Hand Assessment; BE API: Be an Airplane Pilot; BoNT: botulinum toxin; HABIT ILE: Hand and Arm Bimanual Intensive Therapy Including Lower Extremities; MA2: Melbourne Assessment 2.

Each child participated in two visits, one before the intervention (T0) and one at the end of the intervention (T1). Each visit included an assessment of impairment (muscle strength, selectivity, and spasticity), function (Melbourne Assessment 2 [MA2] [21,22]), Assisting Hand Assessment [AHA] [3]), and the 3D bimanual protocol, BE API 2.0. In the HABIT ILE group, visits one and two were conducted the week before and the week after the camp. In the BoNT group, visit one was conducted less than 1 month before the injection and visit two was conducted 4 to 6 weeks after the injection, at the time of peak BoNT effectiveness (Figure 1).

### 2.5. Clinical Assessments

Muscle strength, selectivity, and spasticity were evaluated for 16 muscle groups and individual muscles using the Medical Research Council scale (MRC) [23], the selective motor control (SMC) scale, and the modified Ashworth scale (MAS) [24], respectively. Then, the composite scores of strength, selectivity, and spasticity were calculated according to previous studies [25,26] to express the level of clinical impairment of each child (Supplementary File S1).

The MA2 and AHA were both video-recorded and rated using video-based scoring. The MA2 is a valid and reliable criterion-referenced test of impaired UL movement quality. Four items are rated: (1) range of movement, (2) accuracy of reach and placement, (3) dexterity of grasp, release, and manipulation, and (4) smoothness of movement [21]. The final score is reported as four separate sub-scores, one for each item of movement quality, reported on a scale from 0–100%, where 100% corresponds to full functional capacity. The AHA was used to evaluate the spontaneous use of the impaired UL [3]. It involves the evaluation of 22 bimanual activities classed under the following sub-headings: general use, arm use, grasp and release, fine motor adjustments, coordination, and pace. The total score ranges from 0 to 100 AHA-units; 100 AHA-units indicate normal spontaneous use of the impaired hand. These assessments were rated by trained occupational therapists blinded to the intervention received by the child.

### 2.6. 3D Motion Analysis

#### 2.6.1. BE API 2.0 Protocol

The BE API 2.0 protocol is composed of five bimanual tasks performed during a game scenario designed as “flying missions”. This 3D protocol was previously validated in terms of reliability and validity (content, and discriminative and construct validity) [16]. Each “flying mission” specifically involved either one or two DoF of interest: task 1 “flying over mountains” = elbow extension and wrist adduction, task 2 “slaloming” = external rotation, task 3 “hooking the luggage” = shoulder elevation and humeral plane of elevation, task 4 “opening the door” = wrist extension, and task 5 “refuelling” = elbow supination. Tasks 2, 3, 4 and 5 were asymmetrical, i.e., the movement of interest was only performed by the UL of interest, while the other UL was involved in a different, simultaneous movement. Each asymmetrical task was performed by the impaired UL. The BE API 2.0 protocol is fully described in Cacioppo et al., 2020 [16].

#### 2.6.2. Motion Capture and Data Processing

**Figure 2:**
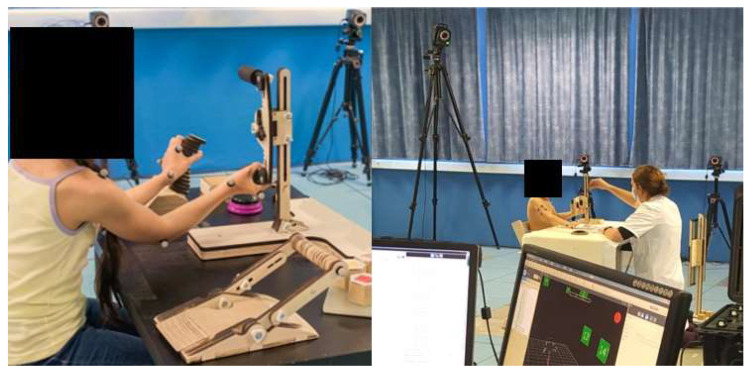
Children performing the “Be an Airplane Pilot” (BE API 2.0) protocol in a motion laboratory with an optoelectronic system and using the game set-up (on the left of the figure).

The data were collected using a 15-camera VICON system at a sampling frequency of 100 Hz (Oxford Metrics Ltd., Oxford, UK). Twenty-six 14 mm reflective markers were fixed on the trunk, arms, forearms, and hands according to the International Society of Biomechanics (ISB) recommendations [27] (Figure 2). The trunk (lateral-flexion, flexion-extension, and rotations), shoulder (elevation, humeral plane of elevation, and rotations), elbow (flexion-extension and pronation–supination), and wrist (flexion–extension and abduction–adduction) angles were calculated using the Euler sequences recommended by the ISB [27]. The shoulder joint was defined as the “thoracohumeral joint”, and its centre was estimated using a functional method [28]. All the data were processed using Matlab (MathWorks, Natick, MA, USA).

The children performed five consecutive repetitions (five trials) of tasks 1–4 at their own self-selected speed. At the end of the five trials of a given task, the first trial of task 5, “refuelling”, was performed to maintain a fun link between the tasks. For tasks 1, 2, 3, and 4, the first trial was considered a training trial, and the next four trials of each task (four trials × five tasks) were analysed. For each DoF and each task, the mean value of the four trials was calculated for each variable.

#### 2.6.3. Data Analysis

For the kinematic movement patterns, the following variables were calculated:

- The Arm Variable Scores (AVS) [29] were calculated using the root-mean-square error (RMSE) between the point-by-point comparison of each joint angle of each child and the mean value for the same joint angle from a reference database of 20 typically developing children (TDC) included in a previous study [16]. A total of 10 AVS were calculated for each task: the trunk (flexion/extension, abduction/adduction, axial rotation), the shoulder (plane of elevation, elevation, rotation), the elbow (flexion/extension, pronation/supination), and the wrist (flexion/extension, abduction/adduction). The global AVS of each joint angle was calculated by averaging the AVS values for the five tasks.
- The Arm Profile Score (APS) is a kinematic index that reflects the total movement deviation of the UL during each task [29]. It was calculated by averaging the RMSE of the 10 joint angles during each task (10 AVS) and compared to the reference population (TDC group). The global APS was calculated by averaging the APS values for the five tasks.
- The kinematic waveforms of each DoF were calculated by computing the mean angular value of the four cycles at each time point of the time-normalized (0–100%) movement cycles for each task.

The movement quality variables were calculated from the displacement of the wrist joint centre (one value for each task [mean of the four movement cycles per task]):

- Smoothness was estimated using the spectral arc length (SPARC), which measures the arc length of the Fourier magnitude spectrum within an adaptive frequency range [30,31]. Higher SPARC values indicate smoother movement.
- Trajectory straightness was evaluated using the index of curvature (IOC) [32,33]. The IOC corresponds to the distance travelled by the hand divided by the linear distance between the start and end of the movement; therefore, an IOC of one indicates a straight trajectory.

### 2.7. Number of Participants

The number of participants to be included was not based on a statistical calculation but was defined according to the feasibility of inclusions. Considering previous publications involving the BE API protocol [15,16] and other 3D bimanual protocols [9,10,11,12], 20 children were required.

### 2.8. Statistical Analysis

Descriptive statistics were used to report the demographic and clinical data.

The clinical measures and kinematic variables (APS, AVS) were compared before and after the interventions using group and individual analyses. For the group-level analysis (all the children), the data from visits 1 and 2 were compared using the paired Student *t*-test or the Wilcoxon signed-rank test, depending on the variable type, distribution, and normality. For the individual-level analysis, the t-values were calculated to detect the change from pre- to post-intervention for each child. The t-value was calculated as (PT1 − PT0)/√(SET02 + SET12), where PT0 and PT1 are the child’s performance at T0 and T1, and SET0 and SET1 are the standard error of measurement at T0 and T1. This t-value indicates whether the pre- to post-intervention change for a given child reflects a true change or simply a measurement error [34]. After the calculation of the individual t-values, the children were divided into five classes of clinical significance according to the change in APS or AVS: significant decrease (t ≤ 1.96), decrease (1.96 < t < 0), no change (t = 0), increase (0 > t > 1.96), and significant increase (t ≥ 1.96) [35].

The waveform analysis of the joint angles was conducted using Statistical Parametric Mapping (SPM) for one-dimensional data (SPM1d, version 0.4, available for download at https://www.spm1d.org/Downloads.html, accessed on 27 November 2022) using Python 3.9 [36,37]. SPM analyses the entire kinematic waveform by taking the interdependency of data points into account using random field theory; therefore, this method reduces the risk of type I errors. Because of the small size of the dataset, we used non-parametric SPM to test our hypotheses. To evaluate differences in waveforms pre- and post-intervention, a non-parametric paired Hotelling’s T2 test (SnPM{T2}) was performed on the 10 joint angles (trunk, shoulder, elbow, and wrist) for each of the five bimanual tasks. In case of significant differences, a non-parametric post hoc, two-tailed, paired *t*-test (SnPM{t} per joint angle) was conducted. Both group and individual SPM analyses were performed. For all the tests, the number of iterations was set at 10,000. To correct for multiple comparisons on the same study population, we used the Bonferroni-corrected threshold with an alpha of 0.05, which resulted in a corrected alpha of 0.005 (division by the number of comparisons [i.e., 0.05/10]).

The quality of movement parameters (SPARC and IOC) was also compared before and after the interventions at the group and individual levels using the same analysis as for the kinematic variables.

Finally, the correlations between the changes in clinical assessment scores and the motion analysis variables (kinematics and quality of movement) from pre- to post-intervention were explored using a Pearson correlation coefficient, if the scores followed a normal distribution, or a Spearman coefficient otherwise.

All the statistical tests (except the SPM analysis) were performed with a two-tailed significance level of 5% using Jamovi v2.3 (computer software).

## 3. Results

### 3.1. Participants

**Figure 3:**
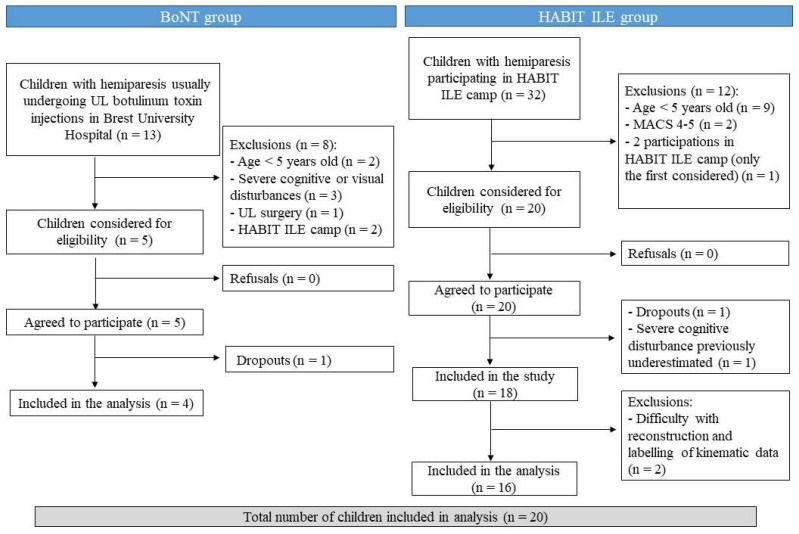
Recruitment flow charts for the children with uCP. HABIT ILE: Hand and Arm Bimanual Intensive Therapy Including Lower Extremities; MACS: Manual Ability Classification System; UL: Upper Limb.

Out of 45 eligible children with hemiparesis, 20 were enrolled in this study (mean age 8.5 [3.9] years; 12 girls; 15 right-side impairment) (Figure 3). Seven children were classed as MACS level I, eight as MACS level II, and five as MACS level III. Sixteen children were included in the HABIT ILE group and four in the BoNT group (Table 1).

### 3.2. Comparison of Clinical Scores Pre- and Post-Intervention

#### 3.2.1. Individual Analysis

The AHA score increased significantly for two children (t ≥ 1.96), tended to increase for eleven (0 > t > 1.96), did not change for one (t = 0), tended to decrease for four (−1.96 < t < 0), and decreased significantly for none (t ≤ −1.96) (two missing data). The MA2 score increased significantly for two children (t ≥ 1.96), tended to increase for ten (0 > t > 1.96), tended to decrease for six (−1.96 < t < 0), and decreased significantly for none (t ≤ −1.96) (two missing data).

#### 3.2.2. Group Analysis (HABIT ILE Group)

Significant increases in muscle strength (MRC pre: mean 62.3 [SD 9.3] vs. post: 65.9 [7.5], *p* = 0.03) and selectivity (SMC pre: 28 [4.8] vs. post: 29.7 [4.0], *p* = 0.01) were found post-intervention in the HABIT ILE group. Spasticity decreased but not significantly (MAS pre: 3.3 [4.3] vs. post: 3.2 [3.6], *p* = 0.87). The mean AHA score increased significantly (AHA pre score: 54.6 [9] vs. post: 57.9 [8.6], *p* = 0.03), whereas the mean MA2 score did not change significantly (MA2 pre score: 59.9 [17.8] vs. post: 67.6 [14.1], *p* = 0.17) (Table 2).

### 3.3. Comparison of Kinematic Movement Patterns of the Impaired UL Pre- and Post-Intervention

#### 3.3.1. Individual Analysis

For each child, 18% to 59% of all the AVS/APS (five tasks) changed significantly post-intervention. Among these changes, between 0% and 38% of the kinematic deviations increased post-therapy, i.e., the AVS/APS values were further from the reference values (TDC) than pre-intervention, and between 5% and 44% of the kinematic deviations decreased, i.e., they were closer to the reference values. Both the proximal and distal kinematic deviations changed for all the tasks for each child. The individual SPM analysis showed that a significant change occurred post-intervention in at least one DoF, mainly for Task 1 (n = 9 children) and Task 2 (n = 10 children).

#### 3.3.2. Group Analysis (HABIT ILE Group)

For the five BE API protocol tasks, the trunk was more flexed post-intervention than pre-. A significant increase in the global trunk flexion–extension deviation was found (Global AVS = 7.1° [2.4] vs. 9.7° [4.8], *p* = 0.03). The Global APS was significantly increased post-HABIT ILE (pre: 22.3° [3.2] vs. post: 23.7° [3.1], *p* = 0.02) (Table 3).

**Figure 4:**
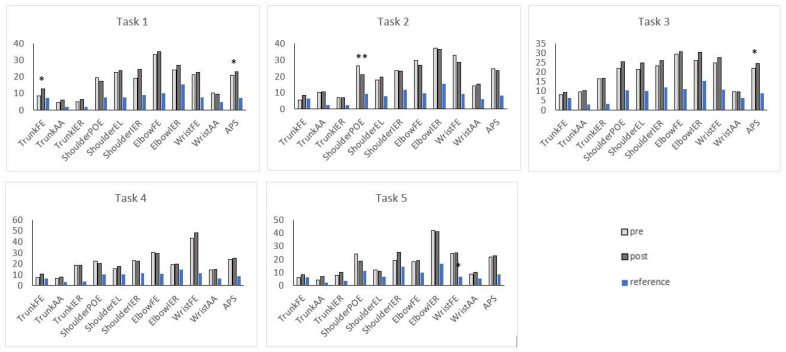
Comparison of APS and AVS of each task pre- and post-intervention. Each bar corresponds to one Arm Variable Score (AVS: the deviation index for a joint angle). The height represents the mean movement deviation between the mean value of the children who received HABIT ILE and the mean value for the same joint angle of typically developing children (reference). The Arm Profile Score (APS) for the overall upper limb movement deviation is displayed in a column on the right and is the average movement deviation for all the kinematic variables. Significant differences between both groups are marked with * if *p* < 0.05 and ** if *p* < 0.01. AA: abduction–adduction; APS: Arm Profile Score; EL: elevation; FE: flexion–extension; IER: internal–external Rotation; POE: Plane Of Elevation.

Regarding the individual tasks, the proximal deviations changed significantly in three tasks (Figure 4). In Task 1, the thorax was more flexed post-intervention: the trunk flexion–extension deviation increased significantly (AVS pre: 8.6° [3.7] vs. post: 12.6° [8.6], *p* = 0.02), and the APS increased significantly (APS pre: 20.8° [4] vs. post: 22.8° [3.6], *p* = 0.01). In Task 2, the shoulder was more abducted post-intervention: the shoulder plane of elevation deviation decreased significantly (AVS pre: 26.5° [10] vs. post: 20.9° [9.3], *p* = 0.008). In Task 3, the APS increased significantly (APS pre: 21.8° [4.5] vs. post: 24.4° [3.2], *p* = 0.02). In Task 5, the trunk was more abducted after the UL therapy: the trunk abduction–adduction deviation increased significantly (AVS pre: 3.9° [1.1] vs. post: 6.8° [6], *p* = 0.02).

No other significant changes were found. The SPM analysis showed no significant differences between the pre- and post-intervention waveforms in any task in the HABIT ILE group (Supplementary Figure S1).

### 3.4. Comparison of IOC and SPARC of the Impaired UL Pre- and Post-Intervention

#### 3.4.1. Individual Analysis

No significant changes were found for smoothness (SPARC) or trajectory straightness (IOC) in any task.

#### 3.4.2. Group Analysis (HABIT ILE Group)

No significant changes were found for smoothness (SPARC) or trajectory straightness (IOC) in any task.

### 3.5. Correlations between Changes in Kinematics and Clinical Assessment Scores

In the whole sample, decreased trunk rotation and pronation–supination deviations were correlated with improved AHA scores (r = −0.58, *p* = 0.01; r = −0.61, *p* = 0.007, respectively). Increased trunk abduction–adduction deviations were correlated with improved MA2 scores (r = 0.51, *p* = 0.03).

Stronger correlations were found in the HABIT ILE group: decreased trunk abduction–adduction (r = −0.55, *p* = 0.04), trunk rotation (r = −0.55, *p* = 0.04), and pronation–supination (r = −0.72, *p* = 0.004) deviations were correlated with improved AHA scores. Increased trunk flexion–extension and abduction–adduction deviations were correlated with improved MA2 scores (r = 0.56, *p* = 0.04; r = 0.69, *p* = 0.008, respectively).

No correlations were found between changes in SPARC or IOC values and changes in AHA or MA2 scores.

## 4. Discussion

To our knowledge, the BE-API 2.0 protocol is the first fully validated 3D bimanual protocol for use in children with uCP. It has a high level of reliability and validity [16]. The results of this study showed that the protocol could detect significant changes in kinematic movement patterns after UL interventions (BoNT or HABIT ILE) in children with uCP, both at the individual and group levels. However, no changes were found in UL smoothness or trajectory straightness after the interventions. Moderate to high correlations were found between functional scores (AHA and MA2) and trunk and pronation–supination kinematic deviations after the interventions, suggesting the kinematic changes had an impact on UL capacity and performance.

### 4.1. Different Kinematic Changes Detected in Each Child after the Interventions

At the individual level, the BE API 2.0 protocol detected kinematic changes in all the children irrespective of the type of intervention, but the profiles of the changes differed between the children for each task. These results are in line with the fact that children with uCP have widely differing motor impairments [38] and, consequently, develop different motor strategies for the same goal to compensate for their deficits [25]. Therefore, we suggest that an individual rather than a group approach is more pertinent for the analysis of motor patterns using 3D UL motion analysis. The child’s data should be compared to his/her own previous data. An individual approach using 3D analysis provides specific kinematic information on the UL impairments that negatively affect the child’s movement. This information can be used to formulate an individualized rehabilitation plan according to the child’s goals for upper limb use [39].

### 4.2. Proximal Kinematic Changes Post-Intervention Detected by the Group Level Analysis

This study is the first to assess changes in bimanual function after HABIT ILE using instrumented measurements. A 3D analysis has previously been used to assess the effects of interventions (CIMT [9,40] or HABIT [9,11,14]) on spatiotemporal parameters and kinematics. No changes were found after the CIMT. However, after the HABIT, the goal synchronization time, movement overlap, and time of bimanual movements increased; with regards to the kinematics, the trunk displacement decreased, and the UL joint excursion and elbow extension increased on the impaired side. Similar findings were reported for structured/unstructured practice, although the kinematic variables only improved for structured practice [10].

The BE API 2.0 protocol detected significant changes in trunk and shoulder movement at the group level after HABIT ILE. The standardization of the start position and the fact that changes were not found in all tasks (only in tasks 1, 2, and 5) suggests that these differences may not have resulted from measurement error or the protocol but were likely to be therapy-induced. A previous study involving a 3D unimanual assessment found similar training-induced changes at the proximal joints (scapula and glenohumeral joint), especially during a hand-to-shoulder task [41]. The changes in trunk and shoulder movements found in the present study may result from the fact that HABIT ILE is a bimanual intensive therapy involving tasks that require simultaneous control and coordination of upper and lower limb movements. This trains postural adaptations of the trunk, improving motor control and facilitating task achievement [17,18,42]. Therefore, more trunk movement was induced than in other therapies (ex: HABIT), and significant changes could be expected. Moreover, other studies also reported changes in proximal kinematics after BoNT injections into distal muscles [6]. Therefore, along with other reports of associations between proximal and distal impairments [43,44,45,46], these results suggest that improvements in distal movement could lead to changes in trunk and shoulder mobility. Changes in all the DoF, particularly the distal DoF, were expected since the main goal of the HABIT ILE for the children included was an improvement in hand function (ex: buttoning, cutting food, etc.), and the BoNT injections mostly targeted the distal muscles (e.g., flexor carpi radialis and ulnaris). However, the kinematic model used in this study was not designed to analyse hand and finger kinematics [47], which may have been more specifically improved by these therapies than wrist and elbow kinematics.

### 4.3. No Change Detected in Quality of Movement Post-Intervention

No significant changes were found in the quality of movement after either HABIT ILE or BoNT. Movement quality cannot be easily measured in clinical practice; the objective analysis provided by the BE API 2.0 protocol thus complements the clinical assessment of UL movements. The lack of change found after the interventions could result from several factors. First, the SPARC may be insufficiently responsive to changes in UL movement in children with uCP. Studies have found improvements in trajectory straightness in 3D unimanual tasks after unimanual training (using the IOC) [41], as well as in smoothness [47] but using other smoothness metrics, and the effects mainly occurred in the long term.

The SPARC is a valid smoothness metric that was found to be responsive to change in reach-to-point and reach-to-grasp tasks performed in different conditions (e.g., different movement durations and distances) in adults with stroke [31,48]. However, its responsiveness to change after an intervention has only been evaluated in adult gait: no significant change occurred in the SPARC despite changes in another smoothness metric [49]. The responsiveness of the SPARC has not previously been assessed in children with uCP. A second explanation for the lack of change in the SPARC is that the standardized and highly reliable BE API 2.0 protocol [16] limited the expression of changes in smoothness and trajectory straightness, despite the changes in the kinematic variables. A third explanation could be that neither HABIT ILE nor BoNT therapy induced changes in the quality of movement, especially the HABIT ILE. This is supported by the fact that previous studies that measured smoothness using clinical assessments found no change in smoothness after HABIT ILE [18].

### 4.4. Relationship between Changes in Movement Deviations and Function

The changes in kinematic variable values were correlated with the changes in the gold standard AHA and MA2 scores; this demonstrates that movement impairments can be objectively characterised and contributes to understanding clinical improvements in movement impairments. Another study found an association between improvement in trajectory straightness and reduced spasticity in the UL flexor muscle chain after unimanual therapy (CIMT) [41], highlighting the negative impact of neuromotor disorders on UL kinematics. To our knowledge, no studies have explored the relationship between kinematic improvements in an impaired UL and functional scores in children with uCP. In this study, the decrease in trunk flexion and pronation–supination deviations after therapy was related to improved clinical bimanual performance scores; this demonstrates the extent of the contribution of these DoF to bimanual task performance. Despite the fact the trunk deviation remained higher than the reference data, the unimanual capacity was improved. Postural adjustments are particularly important during bimanual movements to ensure trunk stability, which facilitates bimanual coordination in children with an impaired UL [50].

### 4.5. Limits

This study may have been underpowered because of the small number of participants, which could explain the lack of significant changes in some of the group analyses. The risk of type I errors was low because we applied a Bonferroni correction on all the UL DoF (x10) results for each task in the SPM analysis; however, this may have led to an underestimation of other kinematic changes that may have been clinically relevant. The children included had different UL rehabilitation objectives; therefore, the content of the interventions (HABIT ILE or the muscles targeted by BoNT injections) varied somewhat, which may have influenced the responsiveness of the BE API 2.0 protocol at the individual and group levels. The AHA score only changed significantly for a few children; therefore, we were unable to calculate the minimal clinically important difference for the kinematic and movement quality variables using this reference anchor.

### 4.6. Perspectives

New data analysis methods, such as deep learning technologies, are emerging and could help to enhance the performance of quantitative assessment methods [51]. Recently, clustering analysis was used to determine different UL patterns in children with uCP [25]. Quantitative data-driven approaches can be used to provide more specific information regarding movement execution and thus help clinicians to determine individualized, goal-directed therapeutic interventions.

## 5. Conclusions

The BE-API 2.0 protocol demonstrated responsiveness to change at the individual and group levels after UL interventions (BoNT or HABIT ILE) in children with uCP. The protocol detected changes in the proximal UL kinematics after the interventions, but no changes were found in the quality of movement. The changes in trunk and pronation–supination kinematics were related to the changes in bimanual and unimanual capacity found by clinical assessments. These data provide new information on movement abnormalities in children with uCP and demonstrate the complementarity of 3DMA to clinical assessments. The analysis of kinematic changes induced by different UL interventions is important to increase understanding of the effects of specific interventions. The inclusion of a hand model in the protocol would be useful to detect changes in dexterity and grasp. The BE API 2.0 protocol is now fully validated and can be implemented in clinical practice. The use of this protocol will allow clinical decisions to include changes in kinematic patterns as part of an individualised approach to treatment.

## Figures and Tables

**Table 1:** Description of study participants.

Inclusion No.	Age (years)	Sex	Pathology	Cerebral Lesion	Impaired Side	MACS Level	Therapy	Therapy Modalities
1	9	F	Stroke	Haemorrhagic stroke (at 7 yo.)	Left	3	HABIT ILE	65 h
2	6	F	CP	Ischaemic stroke	Right	1	HABIT ILE	65 h
3	5	F	CP	Haemorrhagic stroke	Left	1	HABIT ILE	65 h
4	7	M	CP	Ischaemic stroke	Right	1	HABIT ILE	65 h
5	5	M	CP	/	Right	2	HABIT ILE	65 h
6	6	F	CP	Ischaemic stroke	Right	2	HABIT ILE	65 h
7	8	F	CP	Leukomalacia	Right	1	HABIT ILE	65 h
8	7	F	CP	Ischaemic stroke	Right	2	HABIT ILE	65 h
9	6	M	CP	Ischaemic stroke	Right	1	HABIT ILE	65 h
10	6	F	CP	Ischaemic stroke	Right	3	HABIT ILE	65 h
11	6	M	CP	Ischaemic stroke	Right	2	HABIT ILE	65 h
12	16	F	CP	Ischaemic stroke	Right	2	BoNT	Triceps Brachii
13	14	F	CP	Ischaemic stroke	Left	2	BoNT	Pronators, ulnar & radial flexors, thumb opposors
14	14	M	CP	Ischaemic stroke	Right	3	BoNT	Biceps Brachii, pronators
15	5	F	CP	Schizencephaly	Right	1	HABIT ILE	30 h
16	6	M	CP	Ischaemic stroke	Right	1	HABIT ILE	65 h
17	7	F	CP	Malformation	Right	2	HABIT ILE	65 h
18	7	F	CP	/	Right	3	HABIT ILE	65 h
19	12	M	Stroke	Ischaemic stroke (at 10 yo)	Left	3	HABIT ILE	65 h
20	17	M	CP	Ischaemic stroke	Left	2	BoNT	Biceps Brachii, pronators, ulnar & radial flexors

BoNT: Botulinum toxin injections; CP: cerebral palsy; F: female; HI: Hand and Arm Bimanual Intensive Therapy Including Lower Extremities; M: male; MACS: Manual Ability Classification System; yo: years old.

**Table 2:** Results of clinical assessments pre- and post-intervention (HABIT ILE group).

HABIT ILE Group	Pre-Intervention (n = 16)	Post- Intervention (n = 16)	Mean Difference [SD]	*p*-Value	Cohen’s d
**Clinical outcomes**					
Strength (/80)	62.3 [9.3]	65.9 [7.5]	3.5 [5.6]	0.03	−0.63
Spasticity (/80)	3.3 [4.3]	3.2 [3.6]	−0.1 [3.1]	0.87	0.04
Selectivity (/32)	28.0 [4.8]	29.7 [4.0]	1.8 [2.2]	0.01	−0.96
**Functional outcomes**					
AHA (AHA-units/100)	54.6 [9]	57.9 [8.6]	3.3 [4.9]	0.03	−0.67
MA2 (%)	59.9 [17.8]	67.6 [14.1]	7.7 [18.1]	0.17 W	−0.43

Data are mean [SD]. Comparison with *t*-test or Wilcoxon rank (W). AHA: Assisting Hand Assessment; HABIT ILE: Hand and Arm Bimanual Intensive Therapy Including Lower Extremities; MA2: Melbourne Assessment Test 2.

**Table 3:** Comparison of global AVS and APS pre–post-intervention (all tasks).

	Global Deviations	Pre-Intervention (°)	Post- Intervention (°)	Mean Difference [95% CI]	*p*-Value	Effect Size
HI group	Trunk FE	7.1 [2.4]	9.7 [4.8]	2.7 [0.4–5]	0.03	−0.62
	Trunk AA	6.9 [1.6]	8.2 [4.2]	1.2 [−0.8–3.2]	0.25 ^W^	−0.34
	Trunk IER	10.7 [2.7]	11.8 [3.5]	1.3 [−0.8–3]	0.24	−0.31
	Shoulder POE	22.5 [6.4]	20.5 [4.9]	−2 [−5.4–1.3]	0.22	0.32
	Shoulder El	17.7 [5.6]	19.1 [5.3]	1.4 [−0.5–3.4]	0.14	−0.40
	Shoulder IER	21.5 [6.5]	24.2 [8.9]	2.7 [−1.4–6.9]	0.43 ^W^	−0.24
	Elbow FE	27.8 [7.2]	28.3 [5.9]	0.5 [−2.5–3.6]	0.71	−0.09
	Elbow IER	29.9 [8.1]	31.1 [9.5]	1.2 [−1–3.3]	0.26	−0.30
	Wrist FE	27.7 [9.2]	30.3 [12.7]	2.6 [−1.3–6.5]	0.50 ^W^	−0.21
	Wrist AA	11.6 [3.7]	12 [4.6]	0.4 [−1.5–2.2]	0.63 ^W^	0.15
	APS	22.3 [3.2]	23.7 [3.1]	1.4 [0.3–2.5]	0.02	−0.68

Data are mean [SD] unless otherwise stated. Significant differences between sessions were analysed using the *t*-test or Wilcoxon test [W]. AA: abduction–adduction; APS: Arm Profile Score; AVS: Arm Variable Score; EL: elevation; FE: flexion–extension; IER: internal–external rotation; POE: Plane Of Elevation.

## Data Availability

The data presented in this study are available on request from the corresponding author. The data are not publicly available due to hospital privacy.

## Supplementary Materials

The following supporting information can be downloaded at: https://www.mdpi.com/article/10.3390/s23094235/s1, Supplementary File S1: Scales used for assessment of muscle strength, motor selectivity, and spasticity scores; Supplementary Figure S1: SPM analysis for all tasks of the BE API protocol (group level).
