# Supplementary material for: Detecting Movement Changes in Children with Hemiparesis after Upper Limb Therapies: A Responsiveness Analysis of a 3D Bimanual Protocol
Source: Sensors (Basel). 2023 Apr 24;23(9):4235. doi: 10.3390/s23094235 (PMC10181373; doi:10.3390/s23094235)
Supplement: Supplementary file 1 [file sensors-23-04235-s001.zip › sensors-2313714-supplementary/Figure S1.pdf]

Figure S1. SPM analysis for all tasks of the BE API protocol (group level)

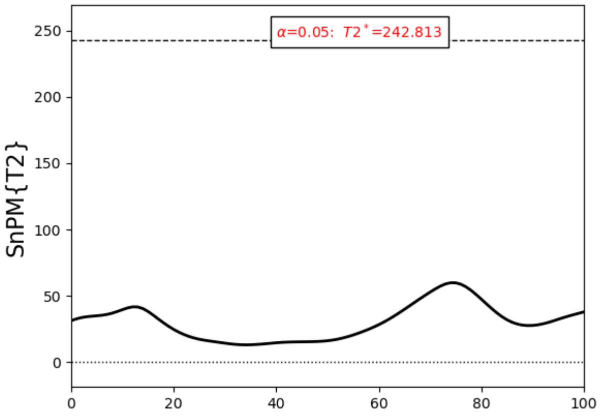

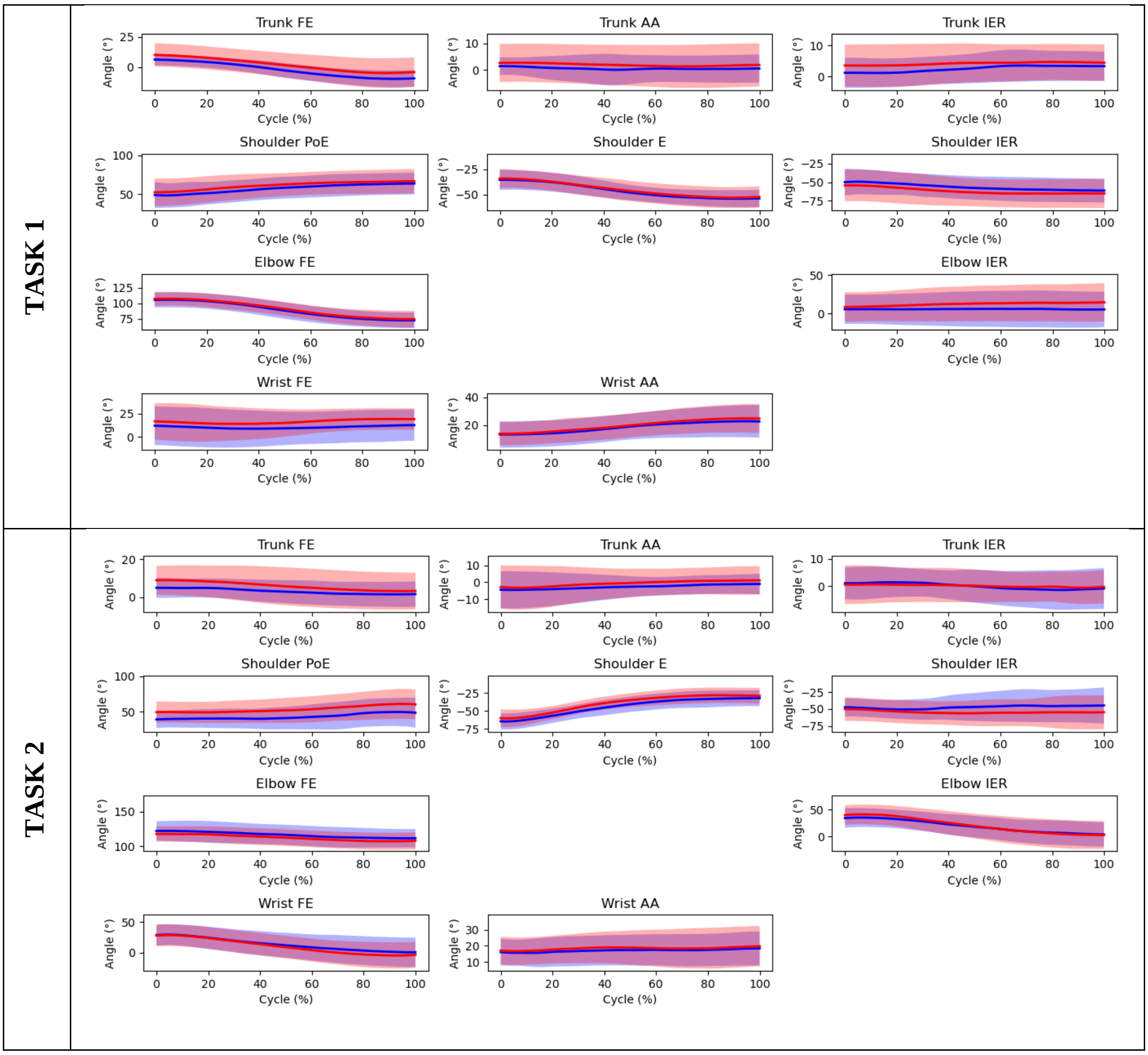

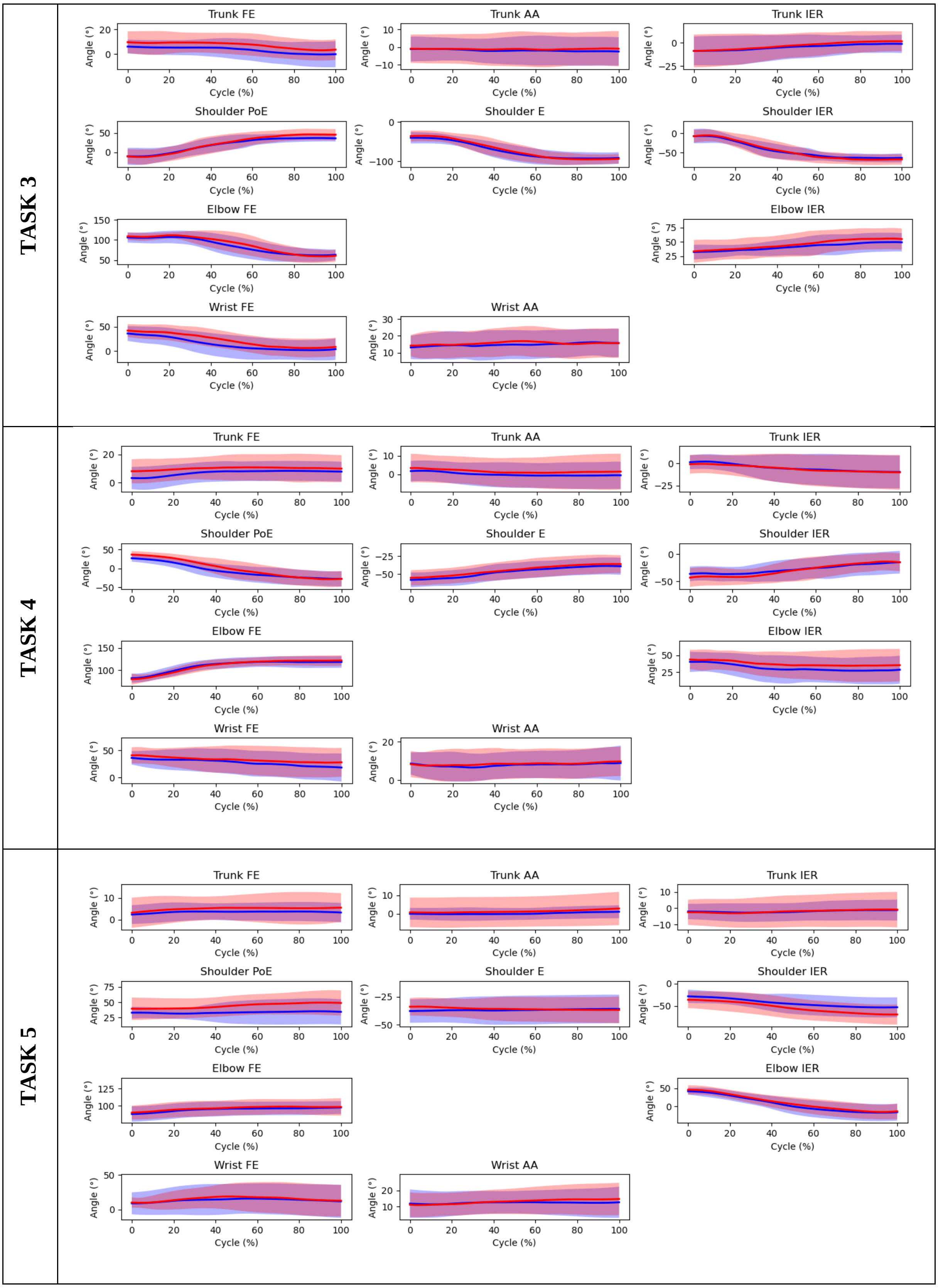

The waveform analysis of joint angles was conducted with the Statistical Parametric Mapping (SPM) for 1 dimensional data toolbox (SPM1d, version 0.4, available for download at https://www.spm1d.org/Downloads.html). SPM1d allows hypothesis testing over the entire spectrum by considering the interdependency of the data points using random field theory and thus reduces the risk of type I errors. For every joint angle, the

waveforms were compared with the conventional univariate statistic, outputting a statistical curve (F-curve). Next, random field theory was applied to estimate the critical threshold above which only 5% of equally smoothed random data was expected to cross ($\alpha < 0.05$). When clusters (i.e., differences between pre and post therapy) were identified, the location, extent, and a single p-value were extracted. Clusters smaller than 5% of the movement cycle are not reported because of their low clinical relevance. For the SPM1d post-hoc comparisons, we used the Bonferroni-corrected threshold with an alpha of 0.05 which resulted in a corrected alpha of 0.0051 (division by the number of comparisons [i.e. 0.05/10]).
