# Supplementary material for: Detecting Movement Changes in Children with Hemiparesis after Upper Limb Therapies: A Responsiveness Analysis of a 3D Bimanual Protocol
Source: Sensors (Basel). 2023 Apr 24;23(9):4235. doi: 10.3390/s23094235 (PMC10181373; doi:10.3390/s23094235)
Supplement: Supplementary file 1 [file sensors-23-04235-s001.zip › sensors-2313714-supplementary/Supplementary File S1.pdf]

Supplementary File S1. Scales used for assessment of muscle strength, motor selectivity and spasticity scores

Muscle strength, selectivity and spasticity were evaluated for 16 muscle groups and individual muscles. Muscle strength was evaluated with the Medical Research Council scale (MRC), a six-point ordinal scale from 0 to 5 in which 0 indicates no muscle contraction and 5 indicates normal strength. A composite strength score was defined as the sum of the muscle weakness scores (total score: 0–80). Muscle selectivity was evaluated with the Selective Motor Control (SMC) scale, a five-point ordinal scale from 0 to 2, rated in increments of 0.5, in which a score of 2 corresponded to normal selectivity. A composite selectivity score was defined as the sum of the muscle selectivity scores (total score: 0–32). Muscle spasticity was evaluated with the Modified Ashworth Scale (MAS), a six-point ordinal scale, rated from 0 to 4 (possible scores: 0,1,1+,2,3 and 4). A score of 0 corresponds to normal muscle tone, whereas a score of 4 corresponds to no possible movement due to rigidity. A composite spasticity score, rated from 0 to 5, was defined as the sum of the MAS scores for each child (total score: 0–80 [16 muscles × 5 levels]).

<u>Medical Research Council (MRC) Scale for Muscle Strength [1]</u>

0 = No movement;
1 = Only a trace or flicker of movement is seen or felt, or fasciculations are observed;
2 = Muscle can only move if resistance of gravity is removed;
3 = Strength further reduced such that joint can be moved only against gravity with examiner's resistance completely removed;
4 = Strength reduced, but contraction can still move joint against resistance;
5 = Muscle contracts against full resistance

<u>Selective Motor Control (SMC) scale</u>

0 = unable, minimal contraction
0,5 = contraction and minimal movements and/or a lot of co-contractions
1 = impaired MSC, dissociated control, limited movement, possible co-contractions
1,5 = MSC correct but lack of fluidity or limitation in movement (co-contractions)
2 = normal MSC, contraction and appropriate muscles

<u>Modified Ashworth Scale (MAS) [2]</u>

0: No increase in muscle tone
1: Slight increase in muscle tone, manifested by a catch and release or by minimal resistance at the end of the range of motion when the affected part(s) is moved in flexion or extension
1+: Slight increase in muscle tone, manifested by a catch, followed by minimal resistance throughout the remainder (less than half) of the ROM
2: More marked increase in muscle tone through most of the ROM, but affected part(s) easily moved
3: Considerable increase in muscle tone, passive movement difficult
4: Affected part(s) rigid in flexion or extension

1. *Paternostro-Sluga T, Grim-Stieger M, Posch M, Schuhfried O, Vacariu G, Mittermaier C, Bittner C, Fialka-Moser V, Reliability and validity of the medical research Council (MRC) scale and a modified scale for testing muscle Strength in patients with radial palsy, Rehabil Med 2008; 40: 665–6713.*
2. *Mutlu A, Livanelioglu A, Gunel Mk. Reliability Of Ashworth And Modified Ashworth Scales In Children With Spastic Cerebral Palsy. Bmc Musculoskelet Disord. 2008;9:44.*
